# Prevalence of meconium stained amniotic fluid and its associated factors among women who gave birth at term in Felege Hiwot comprehensive specialized referral hospital, North West Ethiopia: a facility based cross-sectional study

**DOI:** 10.1186/s12884-018-2056-y

**Published:** 2018-10-30

**Authors:** Dagne Addisu, Azezu Asres, Getnet Gedefaw, Simegnew Asmer

**Affiliations:** 1 Department of midwifery, College of medicine and health science, Debre Tabor University, Debre Tabor, Ethiopia; 2 Department of midwifery, College of medicine and health science, Bahir Dar University, Bahir Dar, Ethiopia; 3 Department of midwifery, College of medicine and health science, Wolidia University, Wolidia, Ethiopia

**Keywords:** Meconium, Amniotic fluid, Meconium stained amniotic fluid, Meconium aspiration syndrome, Felege Hiwot

## Abstract

**Background:**

Meconium stained amniotic fluid is one of the risk factors to increase the rate of perinatal morbidity and mortality both in developed and developing countries. Due to a multitude of factors associated with socioeconomic and quality of service, the ill effect of meconium stained amniotic fluid is even worse in developing countries. But very little information is known about the situation in Ethiopia, particularly the study area to design appropriate prevention strategies. Hence, this study aimed to determine the prevalence of meconium-stained amniotic fluid and its associated factors among women who gave birth at term in Felege Hiwot Referral Hospital, North West Ethiopia.

**Methods:**

Institutional based cross-sectional study was conducted at Felege Hiwot Referral Hospital from March 02–May 27, 2018. A total of 495 mothers were included in the study. The study participants were selected by systematic random sampling technique. A combination of chart review and interview were used to collect the data. Data entry and analysis were made by using Epi-data version 3.1 and SPSS versions 23 respectively. Both descriptive & analytical statistics were computed. Statistical significance was considered at *P* < 0.05 and the strength of association was assessed by using adjusted odds ratio.

**Result:**

The prevalence of meconium stained amniotic fluid was found to be 17.8%. Women whose age greater than 30 years [AOR =5.63, 95%CI =3.35–9.44], duration of labor greater than 24 h [AOR = 7.1, 95%Cl =1.67–29.68], induced labor [AOR = 2.60, 95% CI =1.39–4.87], preeclampsia [AOR = 3.45, 95%CI =1.26–9.37] and obstructed labor [AOR =5.9, 95%CI =1.29–29.68] were found to be associated with meconium stained amniotic fluid.

**Conclusions:**

The prevalence of meconium stained amniotic fluid was similar as compared to the international standard. Preeclampsia, maternal age, obstructed labor, induced labor and longer duration of labor were factors associated with an increased risk for meconium-stained amniotic fluid. Thus, early detection and timely intervention are mandatory to decrease prolonged and obstructed labor.

## Background information

The occurrence of meconium-stained amniotic fluid (MSAF) during labor has been long considered the predictor of adverse fetal outcomes such as meconium aspiration syndrome and perinatal asphyxia, which leads to perinatal and neonatal morbidity and mortality [1, 2].

Meconium is a germ-free, thick, black-green, odorless material which is first recognized in the fetal intestine around 12 weeks of gestation and stores in the fetal colon throughout gestation [3, 4].

Passage of meconium in the newborn infants is a developmentally programmed incident; normally occurring within the first 24 to 48 h after birth. However, the fetus may pass meconium in the amniotic fluid during pregnancy due to different reasons. Meconium-stained amniotic fluid is uncommon before 37 weeks of gestation and the occurrence of a meconium-stained amniotic fluid increases with increasing gestational age [1, 5].

Meconium stained liquor (MSL) is the passage of meconium by a fetus in utero during the antenatal period or in labour. According to Royal College of Obstetricians and Gynecologists (RCOG) intrapartum care guideline, meconium stained amniotic fluid is classified as significant MSL and non-significant MSL. Non- significant MSL is defined as a thin yellow or greenish tinged fluid; containing non-particulate meconium whereas significant MSL is explained as dark green or black amniotic fluid that is thick and tenacious and consists lumps of meconium [6].

Meconium stained amniotic fluid contains masses of debris, desquamated cells from the intestine and skin, gastrointestinal mucin, lanugo hair, fatty material from the vernix caseosa and intestinal secretions [4].

The exact etiology of meconium stained amniotic fluid is not clear. However, previous studies suggested that obstetric factors such as (prolonged labour, post-term pregnancy, low-birth weight babies, oligohydramnios, intrauterine growth retardation and hypertensive disorders of pregnancy),medical factors (cholestasis of pregnancy and anemia) and socio-demographic and behavioral risk factors (higher maternal age, maternal drug abuse especially tobacco and cocaine use) are the major contributory factors for the passage of meconium into the amniotic fluid [7, 8].

Evidence showed that the incidence of meconium stained liquor is increasing as the gestational age increases. From 7 to 22% of term pregnancy were complicated by meconium stained liquor worldwide [9, 10].

Meconium-stained amniotic fluid has adverse long and short-term fetal outcomes; especially it increased rates of neonatal resuscitation, respiratory distress, lower Apgar score, neonatal nursery admissions, meconium aspiration syndrome, neonatal sepsis and pulmonary disease [2, 11, 12].

Meconium aspiration syndrome occurs all over the world about 5–10.5% of neonates with MSAF; which accounts around 12% of neonatal mortality (as much as 40% case fatality rate for the neonate and around 2% of perinatal mortality). Furthermore, the rates of severe mental retardation and cerebral palsy are significantly greater among infants born with MSAF [13, 14].

Meconium stained amniotic fluid significantly increase the rate of maternal complications such as meconium-laden amniotic fluid embolism, intrapartum chorioamnionitis, Puerperal endometiritis, wound infection, increased risk of operative delivery and its complication [12, 15].

The perinatal morbidity and mortality related to MSAF can be decreased if major risk factors are recognized early and closely monitoring of the labor and careful decisions are made about the timing and mode of delivery [7].

Even though the magnitude and associated factors of meconium stained amniotic fluid were well studied in the developed countries, there is a paucity of locally generated evidence on the magnitude and associated factors of MSAF to design appropriate prevention strategies in the study area. Therefore, this study was aimed to determine the prevalence of meconium stained amniotic fluid and its associated factors among women who gave birth at term in Felege Hiwot comprehensive Specialized Referral Hospital.

## Methods

### Study settings and design

Hospital based cross-sectional study was conducted at felege Hiwot Comprehensive Specialized Referral Hospital Obstetrics and Gynecology department, Obstetrics ward from March 02–May 27, 2018 GC. This hospital is the only specialized hospital in Bahir Dar town and located in the Amhara region, Bahir Dar special zone, Bahir Dar City. It is located approximately 565 kms North West of Addis Ababa.

Felege Hiwot Comprehensive Specialized Referral Hospital is one of the top ten governmental hospitals in Ethiopia. This Hospital is having around 400 beds & 9 operating tables, serving over 7 million people within its catchment area. The labor ward gives services to around 612 deliveries per month. The Department of Obstetrics and Gynecology has a labor ward with seven beds in first stage room, two delivery couches in the second stage room, four beds in the recovery unit and sixty nine beds in the maternity ward along with two operating rooms. The ward is staffed with five obstetrics and gynecology specialists, thirty three midwives, seventeen clinical nurses, thirty five residents of different years (levels) of study and a varying number of interns.

### Characteristics of participants

All women who gave birth at term in Felege Hiwot Referral hospital were the source population. This study included all women who gave birth at term throughout the day and night during the data collection period. Those mothers who presented with breech presentation and intrauterine fetal death before the onset of labor were excluded from the study.

### Sample size determination

The sample size was determined by taking predictors for MSAF from previous studies and by using Epi info software version 7.2.0.1. After enrolling different significant factors in the previous studies, cord problem was one of the factors which had a maximum number for our sample size [16, 17]. Based on the previous finding, percent of outcome (MSAF) in women with cord problem (exposed group) were 25% and percent of outcome (MSAF) in women without cord problem (unexposed group) were 14.1% [16]. Based on the assumptions of one to one ratio of exposed to unexposed and 95% confidence interval of certainty to have a power of 80%, the sample size was 450. After 10% of non-response rate were added, the final sample size for this study was 495.

### Sampling and sampling procedure

Systematic random sampling was applied to identify study participants from postnatal and maternity ward. To get study participants, first the average numbers of women who delivered during the data collection period was estimated based on the previous delivery, which was obtained by referring a two-month delivery registration book/ record prior to data collection. Totally 1236 women were delivered in two months; on average 618 women were delivered per month. The data were collected within two-month duration. So as to find the sampling fraction, the total number of women who were delivered in two months (1236) was divided by the total number of sample size (495) and it was approximately 3. The first woman was selected by lottery method then every 3rd woman who gave birth was recruited for the study.

Meconium stained amniotic fluid was defined as the presence of meconium in the amniotic fluid which changes the color of the liquor from clear to various shades of green, yellow or brownish color depending on the degree of meconium stained liquor.

### Data collection tools and procedures

Data were collected using a combination of interview and chart review by three BSc midwives who were trained for this purpose. Structured interviewer-administered data collection formats were adopted and modified from different kinds of literature. Questionnaires which guided chart review and interview were structured into four logical sections (socio -demographic characteristics, obstetric related factors; medical history and Behavioral related factors). Data on patient specific socio- demographic, obstetric, medical and behavioral information were collected through interview of the mother and by reviewing her medical records.

### Socio-demographic, obstetric, medical and behavioral variables

Socio-demographic, obstetric, medical and other factors were examined as a potential predictor in this analysis. Socio-demographic factors include age, ethnicity, residency, religion, educational status, marital status and occupation. Obstetric related factors include parity, Rh status, the onset of labor, late-term pregnancy, premature rupture of membrane, prolonged premature rupture of membrane, preeclampsia, oligohydramnios, IUGR, antepartum hemorrhage, cord problem, chorioamnionitis, duration of labor, mode of delivery, antenatal care follow up. Medically related factors include diabetes mellitus, gestational diabetes mellitus, anemia, hypothyroidism, hepatitis virus, chronic hypertension, asthma, jaundice, and cardiac disease. Behavioral factors include Cigarette smoking, Cocaine use, marijuana addict and chat chewing.

### Data management and analysis

Data were entered into EPI data version 3.1 then exported to SPSS version 23 for analysis. Descriptive statistics like frequencies and cross tabulations were performed. Multiple logistic regressions were fitted for MSAF and odds ratio (OR) with their 95% confidence interval (95% CI) were calculated to identify associated factors of meconium stained amniotic fluid.

Variables with *p*-values ≤0.2 in bivariate analysis remained in the model as potential confounders for the next level analysis. The Hosmer -Lemeshow goodness-of-fit statistic was used to check if the necessary assumptions for multiple logistic regressions were fulfilled and the model had a *p*-value > 0.05 which proved the model was good.

## Results

### Socio-demographic characteristics

A total of four hundred ninety five women were enrolled in the study with a response rate of 100%. The mean age of the study participants was 28.05 years with standard deviation (SD) of ±5.1 years. Nearly two third, 344 (69.5%) of mothers were in the age group of > 30 years and 403 (81.4%) of them were from urban areas (Table 1).

**Table 1:** Socio-demographic characteristics of women who gave birth at term in Felege Hiwot Comprehensive Specialized Referral Hospital (*n* = 495)

Characteristics	Frequency	Percent (%)
Age		
≤ 30 years	344	69.5
> 30 years	151	30.5
Residence		
Urban	403	81.4
Rural	92	18.6
Ethnic group		
Amhara	473	95.6
Others	22	4.4
Religion		
Orthodox	458	92.5
Muslim	27	5.5
Protestant	10	2
Marital status		
Married/union	467	94.3
Others (Unmarried, single &divorced)	28	5.7
Educational status		
Non-formal education	175	35.4
Primary education	73	14.7
Secondary education	51	10.3
Tertiary education	196	39.6
Occupation		
Merchant	119	24
Governmental/private employee	100	20.2
House wife	249	50.3
Others(student, farmer, daily laborer)	27	5.5

### Obstetrics related characteristics

The mean gestational age and duration of labor were 38.95 weeks and 11.45 h with SD of ±1.276 weeks and ± 5.7 h respectively. Half of, 251 (50.7%) mothers were Para I and 421 (85.1%) had spontaneous onset of labor. The majority (96.4%) mothers had a vertex presentation and the rest were face, brow and cord presentation with 2.63%, 0.61%, and 0.4% respectively. 22 (4.4%) of mothers had preeclampsia and 9 (1.8%) mothers had obstructed labor (Table 2).

**Table 2:** Obstetric characteristics of women who gave birth at term in Felege Hiwot Comprehensive Specialized Referral Hospital (n = 495)

Characteristics	Frequency	Percent (%)
Parity		
Primipara	251	50.7
Multipara	184	37.2
grand multipara	60	12.1
ANC follow up		
Yes	469	94.7
No	26	5.3
Gestational age		
37–40 weeks (term)	426	86.1
41 weeks (late term)	69	13.9
Rh status		
Positive	430	86.9
Negative	65	13.1
APH		
Yes	20	4
No	475	96
Obstructed labor		
Yes	11	2.2
No	484	97.8
IUGR		
Yes	19	3.8
No	476	96.2
Preeclampsia		
Yes	22	4.4
No	473	95.6
Oligohydramnios		
Yes	11	2.2
No	484	97.8
Onset of labor		
Spontaneous	421	85.1
Induced	74	14.9
Duration of labor		
≤ 24 h	485	98
> 24 h	10	2
Mode of delivery		
Spontaneous vaginal delivery	363	73.3
Cesarean section	120	24.2
Instrumental delivery	12	2.4
PROM		
Yes	63	12.7
No	432	87.3

### Medical and behavioral related characteristics

Concerning medical conditions of mothers, seven (1.4%) mothers had anemia, eight (1.6%) had gestational diabetes mellitus (GDM), four (0.8) had asthma and five (1%) of mothers had Hepatitis B virus. Almost all mothers, four hundred ninety five (100%) didn’t use Cocaine, chat, Cigarette smoking and marijuana addict.

### Prevalence of meconium stained amniotic fluid

The prevalence of meconium stained amniotic fluid was found to be 88 (17.8%) with [95% CI = 14.3–21.2]. Out of 88 cases delivered with MSAF, 35 (39.77%) were grade 3 MSAF, 42 (47.73%) were grade 2 MSAF and 11 (12.5%) were grade 1 MSAF.

### Factors associated with meconium stained amniotic fluid

The association between socio-demographic, obstetrical, medical conditions of women, and MSAF were assessed. In the bivariate analysis; maternal age, marital status, Rh status, duration of labor, gestational age, the onset of labor, IUGR, preeclampsia and obstructed labor became significant at 0.2 level of significance. However, maternal age, the onset of labor, preeclampsia, duration of labor and obstructed labor were remained significantly and independently associated with MSAF in the multivariable analysis.

Mothers whose age greater than 30 years were 5.6 times more likely to develop meconium stained amniotic fluid during labor than those less than 30 years [AOR = 5.6, 95%C1 = 3.35–9.44].

Those women with duration of labor > 24 h had about 7.1 times higher odds of developing MSAF than those having less than 24 h [AOR =7.1, 95%Cl =1.67–29.68].

Women who had induced labor were 2.6 times more likely to develop MSAF as compared to the spontaneous onset of labor [AOR = 2.6, 95% CI =1.39–4.87].

Mothers who had preeclampsia were 3.4 times more likely to develop MSAF during labor as compared to those who didn’t have [AOR = 3.4, 95%CI =1.26–9.37].

The chance of developing MSAF in obstructed labor was 5.9 times more likely than those without obstructed labor [AOR = 5.9, 95%CI =1.29–29.68] (Table 3).

**Table 3:** Bivariate and multivariable association of meconium stained amniotic fluid and independent factors among women who gave birth at term in Felege Hiwot Comprehensive Specialized Referral Hospital

Variables	MSAF		COR(95% CI)	AOR(95% CI)
	Yes	No		
Age				
≤ 30 years	34 (9.9%)	310 (90.1%)	1	1
> 30 years	54 (35.8%)	97 (64.2%)	5.0 (3.12–8.25)	5.6 (3.35–9.44)*
Marital status				
Married	79 (16.9%)	388 (83.1%)	1	1
Others	9 (32.1%)	19 (68.9%)	2.3 (1.015–5.33)	2.4 (0.96–6.18)
Rh status				
Positive	72 (16.7%)	358 (83.3%)	1	1
Negative	16 (24.6%)	49 (75.4%)	1.6 (0.87–3.01)	1.5 (0.73–3.07)
Onset of labor				
Spontaneous	64 (15.2%)	357 (84.8%)	1	1
Induced	24 (32.4%)	50 (67.6%)	2.6 (1.53–4.66)	2.6 (1.39–4.87)*
Duration of labor				
≤ 24 h	83 (17.1%)	402 (82.9%)	1	1
> 24 h	5 (50%)	5 (50%)	4.8 (1.37–17.10)	7.1 (1.67–29.68)*
IUGR				
Yes	7 (36.8%)	12 (63.2%)	2.8 (1.087–7.44)	2.4 (0.775–7.70)
No	81 (17.0%)	395 (83.0%)	1	1
Gestational age				
37-40wks	71 (16.7%)	355 (83.3%)	1	1
41wks	17 (24.6%)	52 (75.4%)	1.6 (0.894–2.99)	1.7 (0.896–3.46)
Preeclampsia				
Yes	9 (40.9%)	13 (59.1%)	3.4 (1.43–8.35)	3.4 (1.26–9.37)*
No	79 (16.7%)	394 (83.3%)	1	1
Obstructed labor				
Yes	6 (54.5%)	5 (45.5%)	5.8 (1.007–14.5)	5.9 (1.29–29.68)*
No	82 (16.9%)	402 (83.1%)	1	1

❖ Others mean (unmarried, windowed and divorce)

❖ * means *P* < 0.05

## Discussion

The prevalence of meconium stained amniotic fluid was 17.8% with [95% CI = 14.3–21.2]. This finding was in line with the finding from Jimma University specialized hospital (15.4%) [15]. This might be due to the similarity in socio-demography, health institution and quality of service they provided.

This finding was also in line with the finding from the Nigerian University Teaching Hospital (20.4%) [18]. This might be due to the similarity in accessibility and quality of services.

However, this finding was higher than the study finding in Southeastern Brazil (11.9%) [19] and Israel (10.9%) [20]. This discrepancy might be due to the difference between the accessibility and the quality of services in study settings. In addition to this, this study was done in a tertiary referral hospital which covers a wide catchment area and most of the patients referred to this hospital were already complicated and might have predisposing factors for MSAF.

On the other hand, this finding was lower than the study findings in IPGMER Hospital, India (30.6%) [21]. The difference could be attributed to the time gap between the studies. An additional explanation could be due to the emphasis is given by the Ethiopian government on maternal and child health services in the last years to improving maternal health services program. In addition to this, low behavioral risk factors for MSAF such as smoking, Cocaine use, and Marijuana addict were not found in this study area.

Age of the women was significantly associated with the development of MSAF. This finding was consistent with study findings in Indira Gandhi Medical College [22]. This could be explained as a woman gets older, she is more likely to have a gradual loss of compliance of the cardiovascular vessels that is mainly associated with aging of uterine blood vessels and arterial stiffness which may result in insufficient placental perfusion and in utero fetal hypoxia. This finally leads to passage of meconium into the amniotic fluid.

This study also indicated that a significant association was noted between induced labor and MSAF. This finding was in agreement with study findings in São Paulo, Southeastern Brazil [19]. This might be related to tetanic uterine contraction (uterine tachysystole) following oxytocin administration, which may result in intrauterine fetal hypoxia secondary to inadequate placental perfusion. When the fetus suffers from hypoxia or asphyxia, increased parasympathetic stimulation by vagus leads to passage of meconium.

In this study, longer duration of labor showed a statistically significant association with meconium-stained amniotic fluid. This finding was consistent with the study findings at SRM Medical College [17]. This could be due to a prolonged stressful environment for the fetus, which may result in increased peristalsis of a fetal gastrointestinal tract and relaxation of anal sphincter then the passage of meconium.

Preeclampsia had a statistically significant association with the development of MSAF. This finding was consistent with study findings in Kasturba Hospital Delhi and Somaiya Medical College Mumbai [23, 24]. The reason might be explained by the possibility of placental insufficiency in preeclampsia that leads to intrauterine fetal hypoxia or intestinal ischemia. This intrauterine hypoxia finally weakens the action of rectal sphincters and leading to the passage of meconium.

In this study, obstructed labor had a statistically significant association with the development of meconium-stained amniotic fluid. The finding was in agreement with the study conducted in the Nigerian University Teaching Hospital [18]. The reason might be due to the possibility of maternal dehydration, maternal distress and shock, which may result in intrauterine fetal hypoxia secondary to insufficient placental perfusion then the passage of meconium into the amniotic fluid.

This study shares the limitations of cross-sectional studies and hence may not be possible to establish a temporal relationship between MSAF and explanatory variables. Besides, as the study was conducted in a single referral hospital, the results might not be representative of other institutions and the community. Another limitation is possible to recall bias while determining the gestational age.

## Conclusions

The prevalence of meconium stained amniotic fluid was similar compared to the international standard. Multifaceted factors such as preeclampsia, maternal age greater than 30 years, obstructed labor, induced labor and longer duration of labor were independently associated with an increased risk for meconium-stained amniotic fluid in term pregnancies. Hence, early detection by using a latent follow-up chart and partograph and timely intervention is recommended to decrease prolonged and obstructed labor. We also recommend using induction protocols strictly in a woman who is on induction to prevent uterine tachysystole.

## Abbreviations

AOR: Adjusted Odd Ratio
APH: Ante Partum Hemorrhage
CI: Confidence Interval
COR: Crude Odd Ratio
IUGR: Intra-Uterine Growth Retardation
MAS: Meconium Aspiration Syndrome
MSAF: Meconium Stained Amniotic Fluid
MSL: Meconium Stained Liquor
PROM: Premature Rupture of Membrane
SD: Standard Deviation
